# Supplementary material for: Conservation of C4BP-binding sequence patterns in Streptococcus pyogenes M and Enn proteins
Source: J Biol Chem. 2024 Jun 13;300(7):107478. doi: 10.1016/j.jbc.2024.107478 (PMC11292367; doi:10.1016/j.jbc.2024.107478)
Supplement: Supporting Information [file mmc1.pdf]

Conservation of C4BP-binding Sequence Patterns in *Streptococcus pyogenes* M and Enn Proteins

Piotr Kolesiński, Matthew McGowan, Anne Botteaux, Pierre R. Smeesters, and Partho Ghosh

Supporting Figures S1-S6

Supporting Tables S1-S3

Supporting Figure Legends

Figure S1. Structure of M87^{N100} and its complex with C4BPα1-2. A. Superposition of the two M87 α-helices (orange and wheat, except 80-96 in gray). **B.** Superposition of interfaces between M87^{N00} (aa 79-96) and C4BPα1-2, based on the position of C4BPα1-2. One interface has M87^{N100} in orange and C4BPα1-2 in cyan, and the other has M87^{N100} in wheat and C4BPα1-2 in pale cyan.

Figure S2. C4BP-binding mode of M87 protein. A. Structure of M87 (orange ribbon representation; key side chains in bonds representation, with carbons orange, oxygens red, and nitrogens blue) bound to C4BPα1-2 (cyan ribbon representation; key side chains in bonds representation, with carbons cyan, oxygens red, and nitrogens blue). Hydrogen bonds and salt bridges depicted by dashed black lines. **B.** Electron density (contoured at 2σ, and 2.5 Å from depicted atoms) from a 2mFo-DFc composite omit map for the M87-C4BPα1-2 structure. Only one M87 α-helix is shown for clarity. Coloring as in panel A. **C.** Schematic of C4BP sites (left) and M protein amino acids (right) involved in interaction for the M87 pattern, in open book format. Φ denotes hydrophobic contact, and 'H-b' hydrogen-bonding contact.

Figure S3. Structure and stability of wild-type and mutant M87 proteins.
A. Circular dichroism (CD) spectra of intact wild-type and mutant M87 proteins at 25 °C. The data are an average of two independent measurements. **B.** Thermal melting curves of intact wild-type and mutant M87 proteins, as monitored by the CD mean molar residue ellipticity at 222 nm. The data are an average of two independent measurements.

Figure S4. C4BP-binding mode of M68 protein. A. Structure of M68 (orange ribbon representation; key side chains in bonds representation, with carbons orange, oxygens red, and nitrogens blue) bound to C4BPα1-2 (cyan ribbon representation; key side chains in bonds

representation, with carbons cyan, oxygens red, and nitrogens blue). Hydrogen bonds and salt bridges depicted by dashed black lines. **B.** Electron density (contoured at 2σ, and 2.5 Å from depicted atoms) from a 2mFo-DFc composite omit map for the M68-C4BPα1-2 structure. Coloring as in panel A. **C.** Schematic of C4BP sites (left) and M protein amino acids (right) involved in interaction for the M68 pattern, in open book format. Φ denotes hydrophobic contact.

Figure S5. Structure and stability of wild-type and mutant M68 proteins.

A. CD spectra of intact wild-type and mutant M68 proteins at 25 °C. The data are an average of two independent measurements. **B.** Thermal melting curves of intact wild-type and mutant M68 proteins, as monitored by the CD mean molar residue ellipticity at 222 nm. The data are an average of two independent measurements.

Figure S6. Comparison of C4BP-binding region in different structural states of M87. The ligand-binding site of M87 is shown in cartoon representation, with M87 in its complex with C4BPα1-2 in magenta and with LL-37 in cyan. M87 in its uncomplexed form is in green.

Figure S1

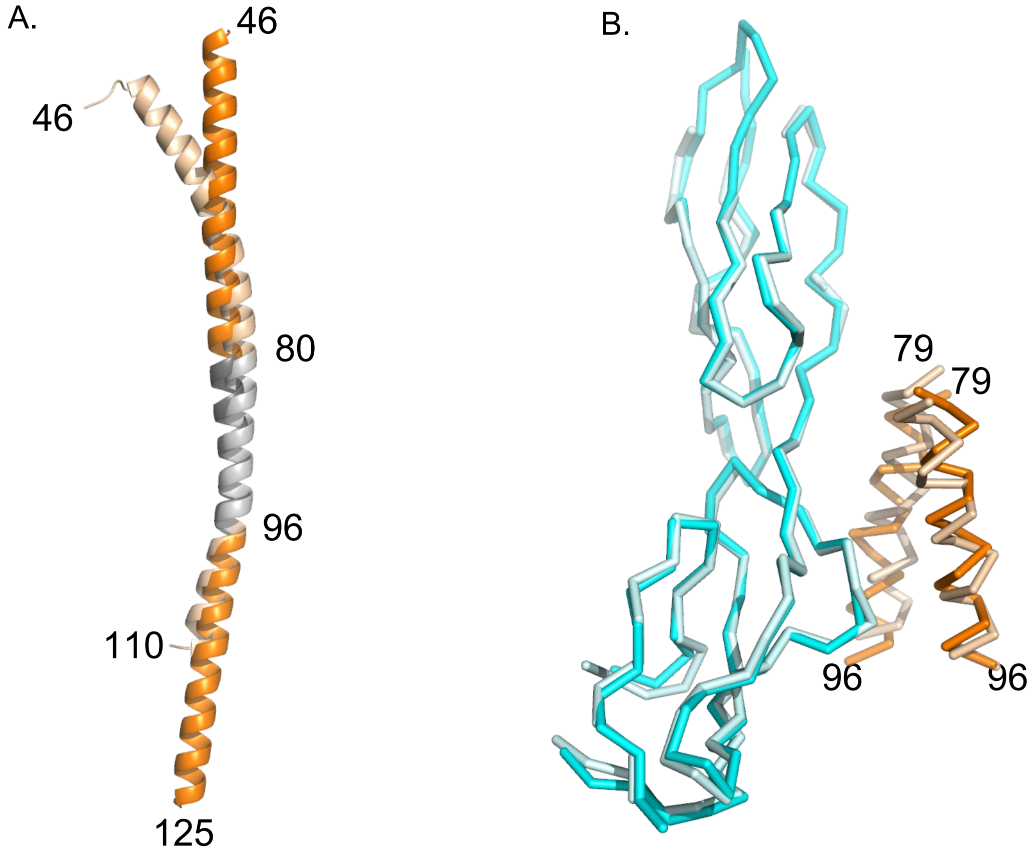

Figure S2

A.

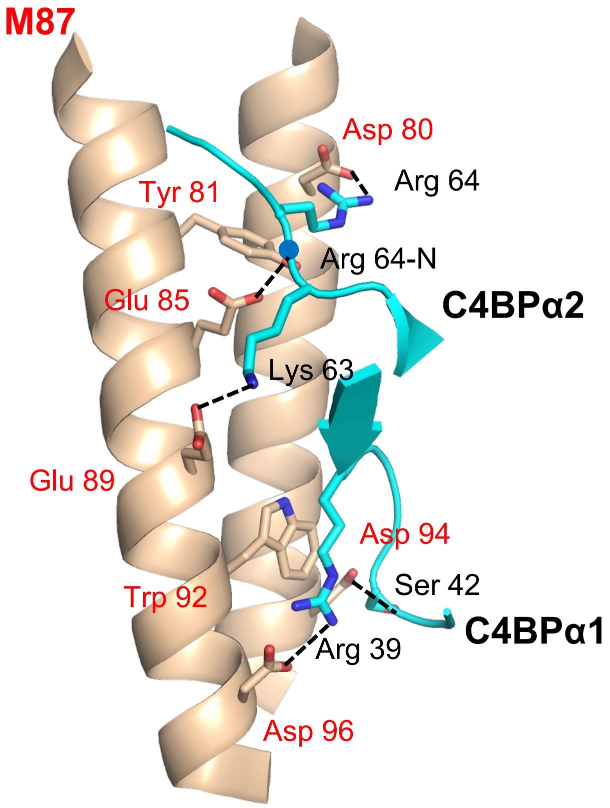

B.

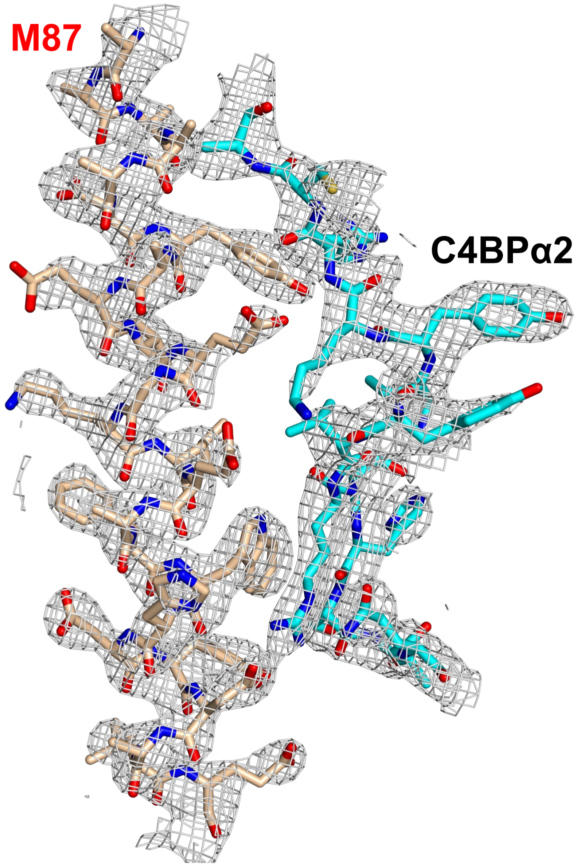

C.

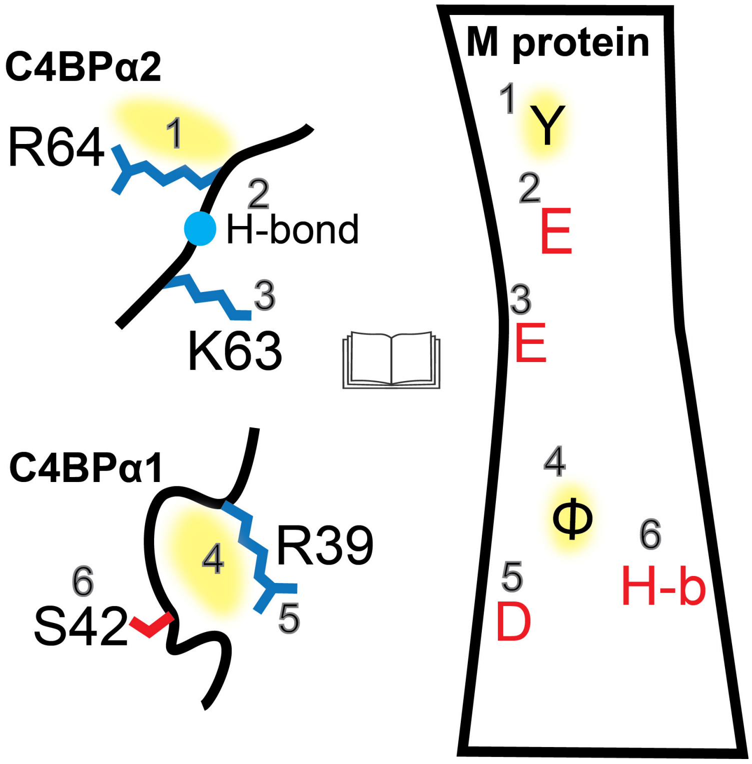

Figure S3

A.

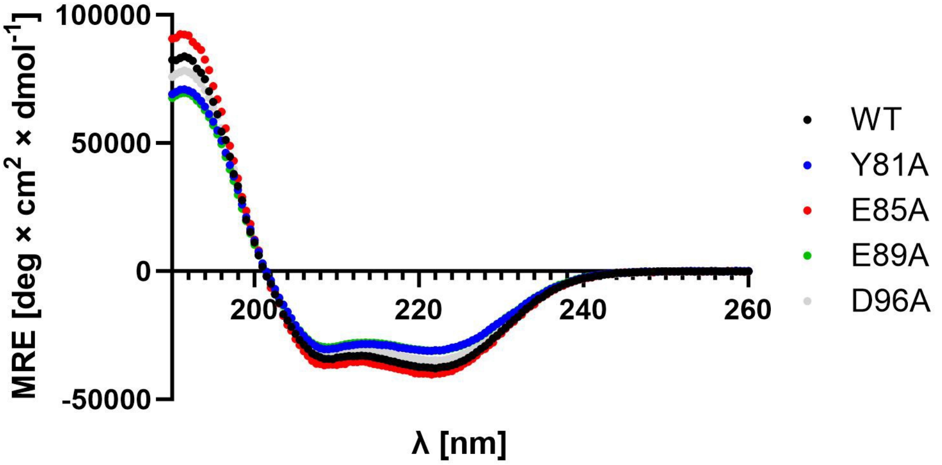

100000
50000
0
-50000
MRE [deg × cm2 × dmol-1]
200
220
240
260
λ [nm]
WT
Y81A
E85A
E89A
D96A

B.

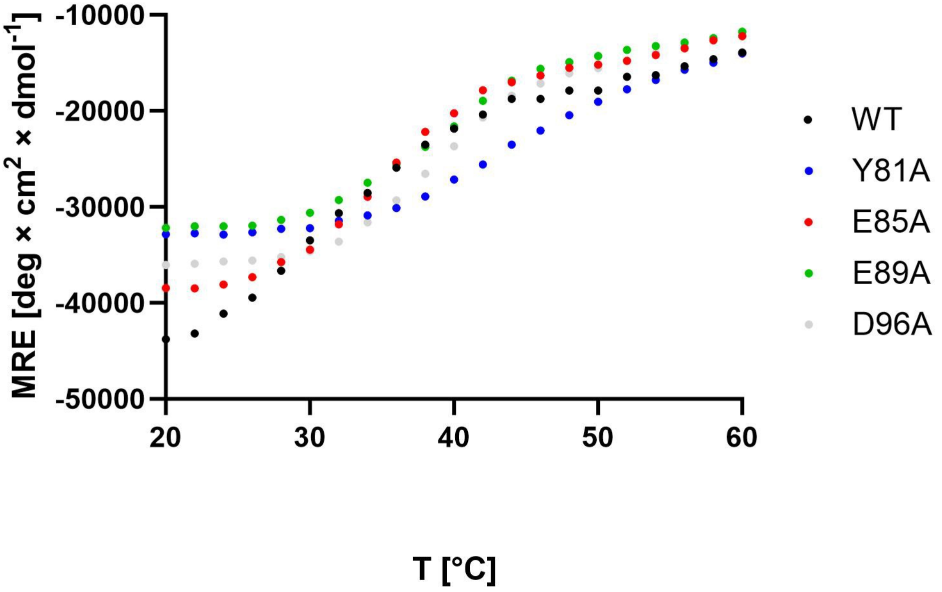

-10000
-20000
-30000
-40000
-50000
MRE [deg × cm2 × dmol-1]
20
30
40
50
60
T [°C]
WT
Y81A
E85A
E89A
D96A

Figure S4

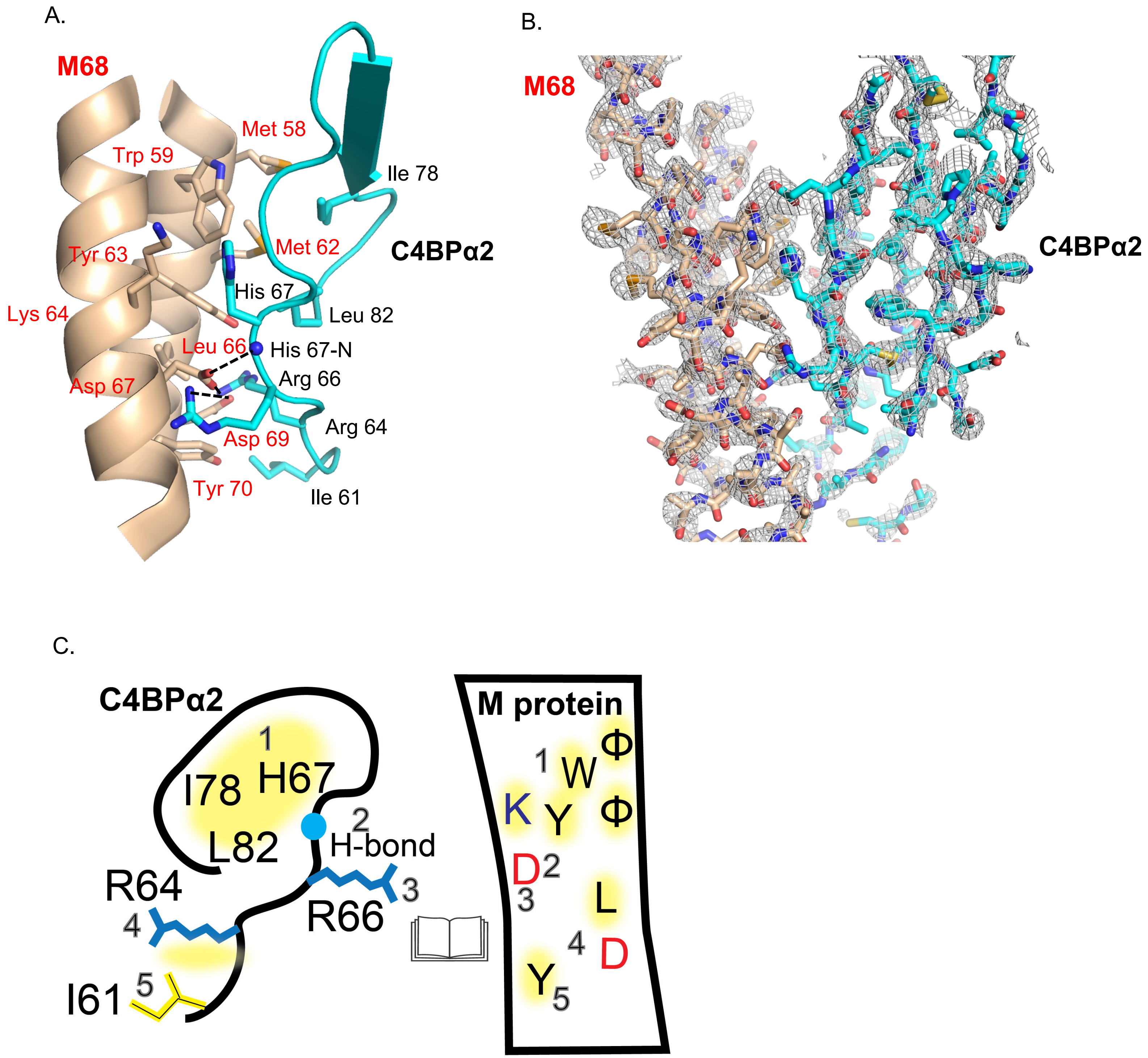

A.
M68
Met 58
Trp 59
Ile 78
Tyr 63
Met 62
C4BPα2
His 67
Lys 64
Leu 82
Leu 66
His 67-N
Arg 66
Asp 67
Arg 64
Asp 69
Tyr 70
Ile 61
B.
M68
C4BPα2
C.
C4BPα2
1
H67
I78
L82
2
H-bond
R64
3
4
R66
5
I61
M protein
Φ
1
W
K
Y
Φ
D
2
3
L
4
D
Y
5

Figure S5

A.

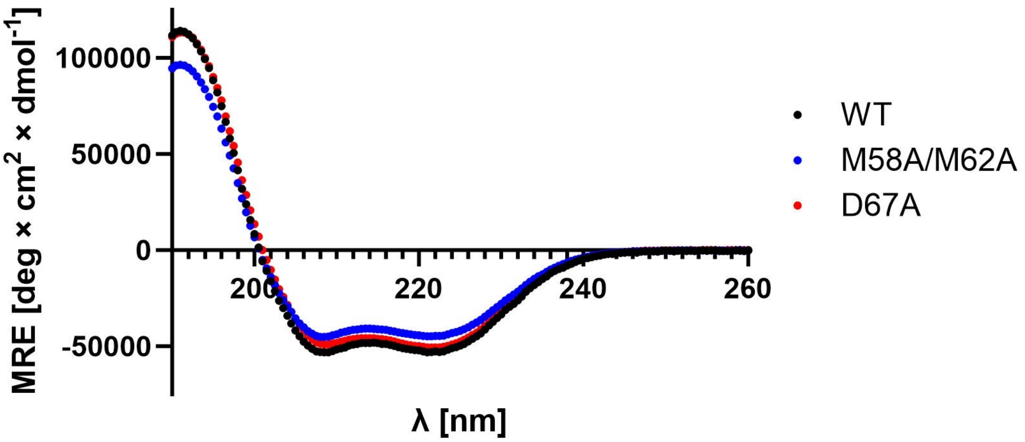
MRE [deg × cm² × dmol⁻¹]
100000
50000
0
-50000
200
220
240
260
λ [nm]
WT
M58A/M62A
D67A

B.

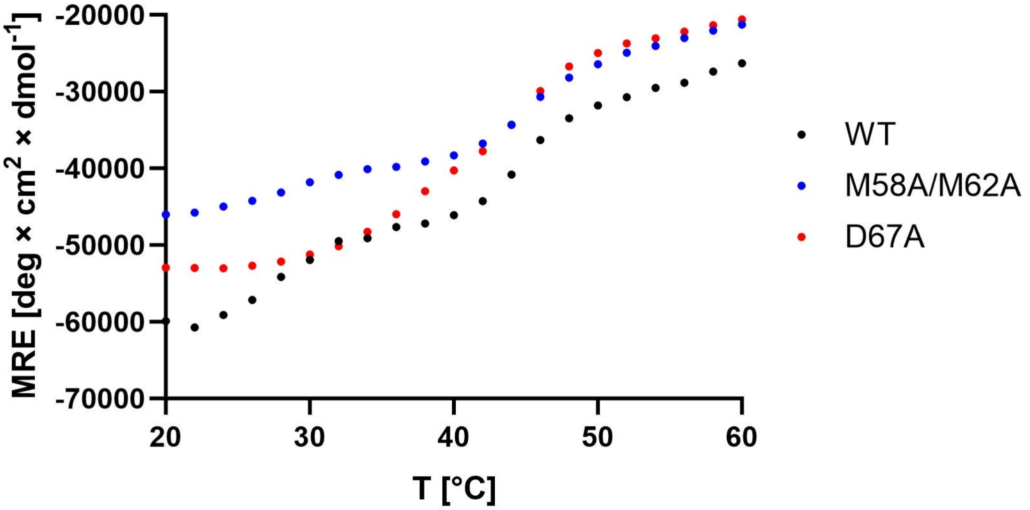
MRE [deg × cm² × dmol⁻¹]
-20000
-30000
-40000
-50000
-60000
-70000
20
30
40
50
60
T [°C]
WT
M58A/M62A
D67A

Figure S6

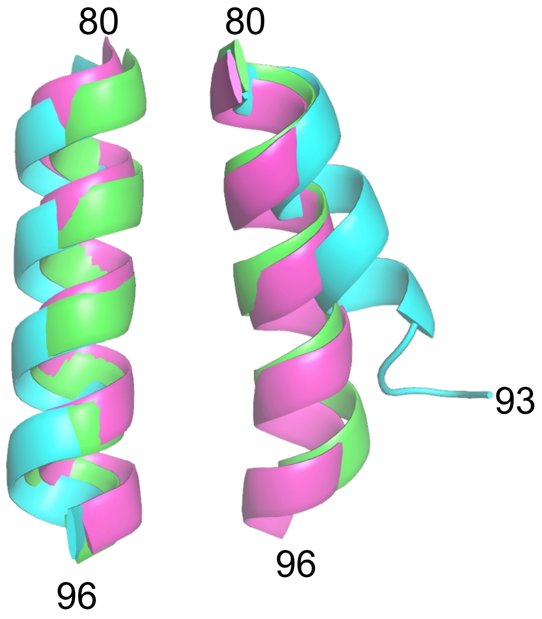

Table S1. Crystallographic Data Collection and Model Refinement

	$M87^{N100}$/C4BPα1-2	SeMet-$M68^{N100}$/C4BPα1-2
**Data Collection**		
Wavelength (Å)	0.98	0.98
Resolution range (Å)	68.81 - 2.69 (2.82 – 2.69)	39.30 – 2.50 (2.60 – 2.50)
Space group	P 2 2_1 2_1	P 2_1 2_1 2
Cell dimensions		
a, b, c (Å)	43.18 73.80 190.40	107.04 49.66 80.40
α, β, γ (°)	90.0 90.0 90.0	90.0 90.0 90.0
Unique reflections	17478 (2045)	15422 (1715)
Multiplicity	6.8 (3.0)	13.1 (13.2)
Completeness (%)	98.64 (87.13)	99.80 (99.80)
Anomalous completeness (%)	N/A	84.65 (66.10)
I/σ(I)	20.3 (1.4)	8.7 (0.8)
Wilson B-factor ($Å^2$)	54.92	43.96
R_{merge}	0.09 (0.953)	0.275 (3.524)
$CC_{1/2}$	0.997 (0.452)	0.997 (0.650)
**Refinement**		
Resolution range (Å)	68.81 - 2.69 (2.82 – 2.69)	37.63 – 2.50 (2.60 – 2.50)
No. of reflections (work/test set)	17414/872	13083/1327
R_{work}/R_{free}	0.24/0.28 (0.35/0.37)	0.31/0.34 (0.39/0.46)
No. of non-hydrogen atoms	2705	1632
macromolecules	2675	1606
ligands	1	6
solvent	29	20
R.m.s. deviations		
bonds (Å)	0.004	0.002
angles (°)	0.62	0.34
Ramachandran plot		
favoured (%)	97.50	97.71
outliers (%)	0.00	0.00
Rotamer outliers (%)	0.84	0.00
Clashscore	4.11	2.51
Average B-factor ($Å^2$)	56.45	62.58
macromolecules	56.59	62.70
ligands	72.13	52.32
solvent	43.52	64.67
Number of TLS groups	4	3
PDB code	8TCB	8TGT

Supplementary Table S2. C4BP-binding Patterns in M proteins.
List of sequences of M proteins sequences with C4BP-binding patterns. Sequences are ordered by their score, followed by the M type number. Red amino acids are observed or predicted to bind C4BP. Start indicates sequence number (including the signal sequence). M proteins that belong to strains identified as binding C4BP (1) are indicated with bold font. * indicates an M protein shown or predicted to bind LL-37 (2). A horizontal line, if present, indicates a threshold set by an M protein from a strain that does not bind C4BP (e.g., M24 or M12) (1).

M87 Pattern

M	Start	Sequence	Score	Clade
**87***	**80**	**DYSEIEGKLEQFWHDYDKLEKE**	**5**	**X**
**112**	**59**	**EIAALQEEKEKLLNELDNVKEE**	**4.7**	**X**
31	98	YLKVLNDWAEKLLKELNGDDVK	4.4	Y
**79***	**79**	**DYSQIEEKLEQFGHDYDKLEKE**	**4.5**	**X**
**103**	**83**	**DYYEIEEKLEQFGRDYDKLEKE**	**4.5**	**X**
**107***	**86**	**GYFNLEDRIEKLGSDYEKLEKE**	**4.5**	**X**
144*	97	GYFNLEDRIEKLGSDYEKLEKE	4.5	X
231	91	GYFNLEDRIEKLGSDYEKLEKE	4.5	X
3	96	YLKGLNDWAERLLQELNGEDVK	4.4	X
**81**	**83**	**ELNEKNRKLEADIADLQDVIED**	**4.4**	**X**
133	62	YLKGLNDWAERLLQELNGEDVK	4.4	Y
**8**	**62**	**LIEENNDLKDKLARNLDLLDNT**	**4.3**	**X**
**9**	**62**	**LYDDYDKLQEKSAEYLERIGEL**	**4.3**	**X**
**82***	**88**	**EYENLEETLQKFGNDYERLENK**	**4.2**	**X**
**90***	**73**	**DYTVLQEAIEGISSENEKLKSE**	**4.2**	**X**
168*	75	YYSLLQETIENMSLENENLDKL	4.2	X
209*	79	DYSQIQEELEQFGHDYDKLEKE	4.2	X
**58***	**80**	**NYETLEKGYDDLEKTLENFGES**	**4.1**	**X**
**58***	**87**	**GYDDLEKTLENFGESYDKLENK**	**4.1**	**X**
180*	59	DYEILEKRYDALEKTLENFGES	4.1	X
31	63	LLERVEQLDNSHNNNYEQYRHY	4	X
34	63	LYDDYNELQDKSAEYLERIGEL	4	X
24	50	TLEKVQERADKFEIENNTLKLK	3.9	Y
88	52	SISNNERLINELTDENNELKDK	3.9	X
19	127	QIQDLESQTQDLESQTQALESQ	3.7	Y
76	58	LYNELQAEHDKLQTKHEELLAE	3.7	X
106	59	IYDELQTKYDELQTKHEELLGE	3.7	X
151	80	ELEERQKNLEKLEHQFQVAADK	3.7	ND

M68 Pattern

M	Start	Sequence	Score	Clade
**68***	**56**	**AKMWENMYKELDRDYSLLEKTV**	**8**	**X**
**55**	**92**	**SNWYATYLKELNDEFEQAYNEL**	**7.7**	**Y**

M2 Pattern

M	Start	Sequence	Score	Clade
**49**	**64**	**EKELYDQIADLTDKNGEYLERIGELE**	**6**	**X**
**102**	**52**	**AAELYDKIKELEEGREELLNDLDKVK**	**6**	**X**
175	59	EAELHDKIADLEEEKAELLNKLDKVE	6	X
31	60	IENLLERVEQLDNSHNNNYEQYRHYS	5.7	Y
34	60	YDKLYDDYNELQDKSAEYLERIGELE	5.7	X
**73**	**58**	**EAELYNKIQELEEGKAELFDKLEKVE**	**5.7**	**X**

**77**	**49**	**DASLHNRITDLEEEREKLLNKLDKVE**	**5.7**	**X**
**84**	**56**	**EEELHNKIADLLDQNEEYLNKIDELK**	**5.7**	**X**
**114**	**65**	**EAELYNKIQELEEGKAELFDKLEKVE**	**5.7**	**X**
**118**	**56**	**VAKLYNQIADLTDKNGEYLERIEELE**	**5.7**	**X**
137	58	LYELYDEITNLEEERGKHLDRIEELE	5.7	X
169	70	HLDLIDNIREKDPQYRALMGENQDLR	5.7	X
232	59	EEKLHNKIADLLDQNEEYLNKIDELK	5.7	Y
**2**	**57**	**EAELHDKIKNLEEEKAELFEKLDKVE**	**5.5**	**X**
**124**	**65**	**EAELHDKIKNLEEEKAELFEKLDKVE**	**5.5**	**X**
95	103	WEQYINSYRDNNDRVQYYLEKANGTD	5.4	Y
27	58	YEELHTKYEELQTKHEELLGENDALK	5.2	X

M22 Pattern

M	Start	Sequence	Score	Clade
**9**	**59**	**YDKLYDDYDKLQEKSAEYLERI**	**5**	**X**
**11**	**93**	**LDKKNKELDSRVTDLIDVIEHD**	**5**	**X**
34	60	YDKLYDDYNELQDKSAEYLERI	5	X
**42**	**99**	**LDKKNKELDSRVTDLIDVIGHD**	**5**	**X**
**44**	**57**	**YDKLSDENDILREKQDEYLTKI**	**5**	**X**
**59**	**84**	**LEKKNKELDSQVAGLIGVVESD**	**5**	**X**
**63**	**82**	**LEKKNKELDSQVAGLIGVVESD**	**5**	**X**
**67**	**57**	**YDKLSKENDILREKQDEYLTKI**	**5**	**X**
**67**	**88**	**LDKKNKELDSQVAGLIGVVESD**	**5**	**X**
**78**	**55**	**IDKLVEENNDLKEERAKYLDLL**	**5**	**X**
**85**	**90**	**LDKKNKELDSRVTDLIDVIEHD**	**5**	**X**
**85**	**108**	**IEHDDQELERKERMYEAFLKQS**	**5**	**X**
**94**	**80**	**LEKKNKELDSQVAGLIGVVESD**	**5**	**X**
**103***	**70**	**FEKQLEDYKKAQKDYYEIEEKL**	**5**	**X**
139	67	YEKLWTEHEKLLGEHGELLGEH	5	X
170	56	VEQLEEEREKLEGEKVELLDKV	5	X
172	65	YESLYDEHEKLYDEKEKLYDEY	5	X
174	53	YDKLHDEYDELQEQNGEYLKKI	5	X
182	106	IEHDDQELERKGRMYEAFLKQS	5	X
191	92	LEKENKELDSQVTDLLDVIEHD	5	X
236	56	YDALRDENTGLRGDQKKLVKKL	5	X
236	87	LEKEKQELENQALNFQDVIETQ	5	X
3	60	IENLLDQVTQLYTKHNSNYQQY	4.7	X
3	148	YQDLDKDFDLAKQGYVLSDKRH	4.7	X
**4**	**59**	**YNALLKENEELKVEREKYLSYA**	**4.7**	**X**
**8**	**59**	**LNDLIEENNDLKDKLARNLDLL**	**4.7**	**X**
**11**	**62**	**YNSLWDENKTLREKQEEYITKI**	**4.7**	**X**
**11**	**111**	**IEHDDQELKRKDRMYEAFLKQS**	**4.7**	**Y**
13	60	YNKLSGDYDKLWDKHGELLSEY	4.7	X
**15**	**63**	**YNKLLDENEKLKEKNEEYLEKI**	**4.7**	**X**
**22**	**59**	**INTLTDENEKLREELQQYYALS**	**4.7**	**X**
**25***	**68**	**VDKLEKQLEGYKKLEEDYFNLE**	**4.7**	**X**
**28**	**62**	**YNTLLTEHEKLRDEYYTLIDAK**	**4.7**	**X**
**42**	**61**	**YNSLWEENKTLREEQEEYIAKI**	**4.7**	**Y**
51	59	LDELYNELHDENSQLVEEKAAL	4.7	X
51	63	YNELHDENSQLVEEKAALLDKL	4.7	X
57	164	YQNTKREYDLIEEELGKKLKEN	4.7	Y
**59**	**57**	**YDALTNENKSLRERDNYLNYL**	**4.7**	**X**
**76**	**59**	**YNELQAEHDKLQTKHEELLAEH**	**4.7**	**X**
**81**	**53**	**YNALWEENEDLRGRERKYIAKL**	**4.7**	**X**
**81**	**102**	**IEDNDQEIKRKDRMYEAFLKQS**	**4.7**	**X**

**85**	**59**	**YNSLWDENKTLREKQGEYITKI**	**4.7**	**X**
**88**	**60**	**INELTDENNELKDKLAQSLDLL**	**4.7**	**X**
**92**	**57**	**YNNLLNEYDDLLAKHGELLSEY**	**4.7**	**X**
**109**	**54**	**YNTLLTENEKLREELQQYYALI**	**4.7**	**X**
**111**	**183**	**IESANSQLEFKNSQISELVAQA**	**4.7**	**Y**
113	58	YNKLSDERNNLLDQNGDLLDQN	4.7	X
133	114	YQDLDKDFDLAKQGYVLSDKRH	4.7	Y
158	59	YNKLSNERDNLLGENGKLWDEN	4.7	X
165	60	YNKLVEENSKLQKQLEEYLDSS	4.7	X
176	66	YNKLVDENDKLQKQLEEYLDSS	4.7	X
177	57	YDALTNENKSLRKERDNYLNYL	4.7	X
193	57	YDRLDEQNHKLVNDNHKLVNDK	4.7	Y
205	61	YNKLLDENEKLKEEIGGYLDKQ	4.7	X
221	193	IESANSQLEFKNSQISELVAQA	4.7	Y
50	56	ISKLYDENSKLIEERADLLGKL	4.5	X
65	58	YSKLLNENDILRDKQDDYLTKI	4.5	X
95	154	LSTLQEELKTAKSVYELAVLST	4.5	Y
134	56	ISKLYDENSKLIEERADLLDKL	4.5	X
157	78	ITDLTTELDEKVASFEAELGRN	4.5	Y
183	55	YSQLHDDYDKLQEQNGEYLKKI	4.5	X
12	195	VDETDRNLQQEKQKVLSLEQQL	4.4	Y
49	50	VEAAENNVSSVARREKELYDQI	4.4	X
55	308	LETINNNLLGNAKDMIAKLSAE	4.4	Y
63	58	YNALTNENKSLRREKDKYLYEK	4.4	X
117	58	YNELSGEYNKLLDQNGNLLDEN	4.4	X
151	43	VEVAENNVSSVARREKELYDQI	4.4	ND
222	63	LNNDLRQLEGKVRNLRSMMHEL	4.4	Y
222	300	LETINNNLLGNAKDMIAKLSAK	4.4	Y
228	181	VDETDRNLQQEKQKVLSLEQQL	4.4	Y
229	197	VDETDRNLQQEKQKVLSLEQQL	4.4	Y
56	55	LSELPGQAQAFSRAFLHEREKN	3.9	Y
56.2	55	LSELPGQAQAFSRAFLHERQKN	3.9	Y

1. Persson J, Beall B, Linse S, Lindahl G. Extreme sequence divergence but conserved ligand-binding specificity in *Streptococcus pyogenes* M protein. PLoS Pathog. 2006;2(5):e47. PubMed PMID: 16733543.
2. Kolesinski P, Wang KC, Hirose Y, Nizet V, Ghosh P. An M protein coiled coil unfurls and exposes its hydrophobic core to capture LL-37. Elife. 2022;11. PubMed PMID: 35726694.

Supplementary Table S3. C4BP-binding Patterns in Enn Proteins.
List of sequences of Enn proteins sequences with C4BP-binding patterns. Sequences are indicated by the M strain followed by Enn protein identifier, and are ordered by the score followed by numerical order of the M type. Red amino acids are predicted to bind C4BP. Start indicates sequence number (including the signal sequence). Enn proteins that belong to strains identified as binding C4BP are indicated with bold font. A horizontal line, if present, indicates a cut-off threshold set by an M protein from a strain that does not bind C4BP (i.e., M24). Enn protein sub-groups (SG) are indicated.

M2 Pattern

Enn	Start	Sequence	Score	SG
**M43_enn172**	**52**	**EAKLHDEIAELLEKNGEYLDKIEELE**	**6**	**SG5**
**M72_enn377**	**52**	**EAKLHDEIAELLEKNGEYLDKIEELE**	**6**	**SG6**
**M74_enn168**	**52**	**EAKLHDEIAELLEKNGEYLDKIEELE**	**6**	**SG5**
**M74_enn173**	**52**	**EAKLHDEIAELLEKNGEYLDKIEELE**	**6**	**SG5**
**M74_enn170**	**52**	**EAKLHDEIAELLEKNGEYLDKIEELE**	**6**	**SG5**
**M116_enn179**	**52**	**EAKLHDEIAELLEKNGEYLDKIEELE**	**6**	**SG5**
**M118_enn58**	**52**	**EAKLHDEIAELLEKNGEYLDKIEELE**	**6**	**SG3**
M24_enn282	58	LYELYDEITNLEEERGKHLDRIEELE	5.7	SG5
M39_enn283	58	LYELYDEITNLEEERGKHLDRIEELE	5.7	SG5
**M41_enn162**	**55**	**DKELHNEIAELLEKNGEYLDKIEELE**	**5.7**	**SG9**
**M41_enn167**	**56**	**DEELHNEIAELLEKNGEYLDKIEELE**	**5.7**	**SG8**
**M43_enn298**	**66**	**YDKLYDDYNELQEKSAEYLERIGELE**	**5.7**	**outlier**
**M91_enn292**	**65**	**YDKLYDDYNELQDKSAEYLERIGELE**	**5.7**	**outlier**

M22 Pattern

Enn	Start	Sequence	Score	SG
**M4_enn2**	**62**	**LEKQKQELENQALNFHDVIETQ**	**5**	**SG3**
**M4_enn14**	**56**	**YDALKDENTGLRGDQTKLVKKL**	**5**	**SG3**
**M4_enn16**	**56**	**YDTLKDENTGLRGDQTKLVKKL**	**5**	**SG3**
**M9_enn33**	**87**	**LEKQKQELENQALNFQDVIETQ**	**5**	**SG3**
**M15_enn36**	**87**	**LEKQKQELENQALNFQDVIETQ**	**5**	**SG3**
**M15_enn37**	**87**	**LEKQKQELENQALNFQDVIETQ**	**5**	**SG3**
**M22_enn151**	**93**	**LEKEKQELENQALNFQDVIETQ**	**5**	**outlier**
**M22_enn342**	**56**	**VEQLEEEREKLEGEKVELLDKV**	**5**	**SG8**
**M28_enn67**	**62**	**LEKQKQELENQALNFQDVIETQ**	**5**	**SG4**
**M33_enn250**	**53**	**YDKLHDEYDELQEQNGEYLKKI**	**5**	**SG5**
**M33_enn249**	**53**	**YDKLHDEYDELQEQNGEYLKKI**	**5**	**SG5**
**M33_enn248**	**53**	**YDKLHDEYDELQEQNGEYLKKI**	**5**	**SG5**
**M43_enn298**	**66**	**YDKLYDDYNELQEKSAEYLERI**	**5**	**SG9**
**M44_enn5**	**87**	**LEKQKQELENQALNFQDVIETQ**	**5**	**SG3**
**M44_enn11**	**87**	**LEKQKQELENQALNFQDVIETQ**	**5**	**SG3**
**M44_enn12**	**87**	**LEKQKQELENQALNFQDVIETQ**	**5**	**SG4**
**M48_enn340**	**56**	**VEQLEEEREKLEGEKVELLDKV**	**5**	**SG8**
**M49_enn40.1**	**56**	**YDALKDENTGLRGDQTKLVKKL**	**5**	**SG3**
**M49_enn40**	**56**	**YDALKDENTGLRGDQTKLVKKL**	**5**	**SG3**
M53_enn230	53	YDKLHDEYDELQEQNGEYLKKI	5	SG5
M53_enn234	53	YDKLHDEYDELQEQNGEYLKKI	5	SG5
**M58_enn48**	**56**	**YDALRDENTGLRGDRTKLVKKL**	**5**	**SG3**
**M58_enn51**	**56**	**YDALRDENTGLRGDRTKLVKKL**	**5**	**SG3**
**M58_enn52**	**56**	**YDALRDENTGLRGDRTKLVKKL**	**5**	**SG3**
**M59_enn240**	**53**	**YDKLHDEYDELQEQNGEYLKKI**	**5**	**SG5**
**M63_enn247**	**52**	**YDKLHDEYDELQEQNGEYLKKI**	**5**	**SG5**
**M68_enn10**	**87**	**LEKQKQELENQALNFQDVIETQ**	**5**	**SG3**
**M68_enn5.4**	**87**	**LEKQKQELENQALNFQDVIETQ**	**5**	**SG3**

M70_enn231	53	YDKLHDEYDELQEQNGEYLKKI	5	SG5
M70_enn232	53	YDKLHDEYDELQEQNGEYLKKI	5	SG5
M73_enn5.1	87	LEKQKQELENQALNFQDVIETQ	5	SG8
M75_enn336	56	VEQLEEEREKLEGEKVELLDKV	5	SG8
M76_enn40	56	YDALKDENTGLRGDQTKLVKKL	5	SG3
M76_enn38	56	YDALKDENTGLRGDQTKLVKKL	5	SG3
M78_enn13	87	LEKQKQELENQALNFQDVIETQ	5	SG3
M78_enn68	87	LEKQKQELENQALNFQDVIETQ	5	SG3
M78_enn14	56	YDALKDENTGLRGDQTKLVKKL	5	SG3
M89_enn26	87	LEKQKQELENQALNFQDVIETQ	5	SG3
M90_enn3	87	LEKQKQELENQALNFQDVIETQ	5	SG3
M90_enn141	93	LEKEKQELENQALNFQDVIETQ	5	SG2
M91_enn292	65	YDKLYDDYNELQDKSAEYLERI	5	SG8
M93_enn237	53	YDKLHDEYDELQEQNGEYLKKI	5	SG5
M97_enn244	52	YDKLHDEYDELQEQNGEYLKKI	5	SG5
M102_enn152	90	LEKEKQELENQALNFQDVIETQ	5	SG2
M104_enn146	93	LEKEKQELENQALNFQDVIETQ	5	SG2
M106_enn155	90	LEKEKQELENQALNFQDVIETQ	5	SG2
M108_enn237	53	YDKLHDEYDELQEQNGEYLKKI	5	SG5
M109_enn17	56	YDALKDENTGLRGDQTKLVKKL	5	SG4
M110_enn28	87	LEKQKQELENQALNFQDVIETQ	5	SG3
M112_enn132	91	LEKEKQELENQALNFQDVIETQ	5	SG2
M114_enn22	87	LEKQKQELENQALNFQDVIETQ	5	SG3
M118_enn60	56	YDALRDENTGLRGDQKKLVKKL	5	SG3
M118_enn60	87	LEKEKQELENQALNFQDVIETQ	5	SG3
M118_enn23	87	LEKQKQELENQALNFQDVIETQ	5	SG3
M124_enn137	93	LEKEKQELENQALNFQDVIETQ	5	SG2
M124_enn138	93	LEKEKQELENQALNFQDVIETQ	5	SG2
M2_enn127	91	LEAINKELNENYYKLQDGIDAL	4.7	SG1
M18_enn300	61	YNKLLDENEKLKEEIGGYLDKQ	4.7	SG9
M46_enn379	59	LDELYNELHDENSQLVEEKAAL	4.7	outlier
M46_enn379	63	YNELHDENSQLVEEKAALLDKL	4.7	outlier
M64_enn306	61	YNKLLDENEKLKEEIGGYLDKQ	4.7	SG9
M80_enn311	61	YNKLLDENEKLKEEIGGYLDKQ	4.7	SG9
M98_enn314	61	YNKLHEENERLKEEIGGYLDKQ	4.7	SG9
M101_enn300	61	YNKLLDENEKLKEEIGGYLDKQ	4.7	SG9
M123_enn300.1	61	YNKLLDENEKLKEEIGGYLDKQ	4.7	SG9
M36_enn255	56	ISKLYDENSKLIEERADLLDKL	4.5	SG7
M43_enn257	56	ISKLYDENSKLIEERADLLDKL	4.5	SG7
M52_enn259	56	ISKLYDENSKLIEERADLLDKL	4.5	SG7
M53_enn252	56	ISKLYDENSKLIEERADLLDKL	4.5	SG7
M53_enn254	56	ISKLYDENSKLIEERADLLDKL	4.5	SG7
M53_enn256	56	ISKLYDENSKLIEERADLLDKL	4.5	SG7

M87 Pattern

Enn	Start	Sequence	Score	SG
M85_enn329	59	YIRQLEEEREKLFDKVDQLEQQ	4.5	SG9
M85_enn330	59	YIRQLEEEREKLFDKVDQLEQQ	4.5	SG9
M2_enn127	101	NYYKLQDGIDALEKEKEDLKTT	4.2	SG1
M8_enn95	110	NYYKLQDGIDALEKEKEDLKTT	4.2	SG1
M8_enn124	55	NYYKLQDGIDALEKEKEDLKTT	4.2	SG1
M9_enn129	101	NYYKLQDGIDALEKEKEDLKTT	4.2	SG1
M25_enn83	104	NYYKLQDGIDALEKEKEDLKTT	4.2	SG1
M25_enn82	104	NYYKLQDGIDALEKEKEDLKTT	4.2	SG1

**M25_enn88**	**104**	**NYYKLQDGIDALEKEKEDLKTT**	**4.2**	**SG1**
**M28_enn121**	**101**	**NYYKLQDGIDALEKEKEDLKTT**	**4.2**	**SG1**
**M60_enn87**	**104**	**NYYKLQDGIDALEKEKEDLKTT**	**4.2**	**SG1**
**M66_enn129**	**101**	**NYYKLQDGIDALEKEKEDLKTT**	**4.2**	**SG1**
**M68_enn101**	**100**	**NYYKLQDGIDALEKEKEDLKTT**	**4.2**	**SG1**
**M73_enn115**	**101**	**NYYKLQDGIDALEKEKEDLKTT**	**4.2**	**SG1**
**M77_enn119**	**101**	**NYYKLQDGIDALEKENEDLKTT**	**4.2**	**SG1**
**M77_enn116**	**101**	**NYYKLQDGIDALEKENEDLKTT**	**4.2**	**SG1**
**M77_enn79**	**104**	**NYYKLQDGIDALEKEKEDLKTT**	**4.2**	**SG1**
**M77_enn81**	**104**	**NYYKLQDGIDALEKEKEDLKTT**	**4.2**	**SG1**
**M77_enn118**	**101**	**NYYKLQDGIDALEKENEDLKTT**	**4.2**	**SG1**
**M77_enn80**	**104**	**NYYKLQDGIDALEKEKEDLKTT**	**4.2**	**SG1**
**M79_enn114**	**103**	**NYYKLQDGIDALEKEKEDLKTT**	**4.2**	**SG1**
**M82_enn107**	**103**	**NYYKLQDGIDALEKEKEDLKTT**	**4.2**	**SG1**
**M87_enn92**	**104**	**NYYKLQDGIDALEKEKEDLKTT**	**4.2**	**SG1**
**M92_enn97**	**100**	**NYYKLQDGIDALEKEKEDLKTT**	**4.2**	**SG1**
**M92_enn100**	**100**	**NYYKLQDGIDALEKEKEDLKTT**	**4.2**	**SG1**
**M103_enn102**	**100**	**NYYKLQDGIDALEKEKEDLKTT**	**4.2**	**SG1**
**M117_enn120**	**101**	**NYYKLQDGIDALEKEKEDLKTT**	**4.2**	**SG1**
**M43_enn298**	**68**	**LYDDYNELQEKSAEYLERIGEL**	**4**	**SG9**
**M91_enn292**	**68**	**LYDDYNELQDKSAEYLERIGEL**	**4**	**SG8**
**M111_enn158**	**57**	**IYEELETKYEELQTKHEELLGE**	**4**	**outlier**
M64_enn306	77	GYLDKQEQLEQLERQYQIAADK	3.7	SG9
M80_enn311	77	GYLDKQEQLEQLERQYQIAADK	3.7	SG9
